# Functionalized Silica Fume for Efficient Cd^2+^ Removal from Aqueous Solutions

**DOI:** 10.3390/molecules30204141

**Published:** 2025-10-21

**Authors:** Jianeng Zhu, Kuixian Wei

**Affiliations:** 1 Faculty of Metallurgical and Energy Engineering, Kunming University of Science and Technology, Kunming 650093, China; zhujn2019@126.com; 2 Kunming General Survey of Natural Resources Center, Kunming 650111, China

**Keywords:** silica fume, surface functionalization, activation, amination, coordination mechanism

## Abstract

The rapid development of the silicon industry has led to the massive production of silica fume (SF), while its improper disposal poses environmental risks and represents a waste of resources. Unlike conventional methods that require dissolution and high-pressure treatment, this study pioneers a low-cost direct-functionalization strategy that preserves the inherent microsphere structure of SF, using calcination for carbon removal and activation for surface hydroxylation. The activated SF was synthesized into TACA-APTES-SF by reacting with 3-aminopropylethoxysilane (APTES) and 1,3-Thiazole-2-carbaldehyde (TACA). SEM, FT-IR, and XPS were used to characterize these samples, revealing amino groups and sulfur groups grafted onto the SF surface successfully. The adsorbent demonstrated highly effective adsorption of Cd^2+^ ions. Throughout five reuse cycles, TACA-APTES-SF maintained a high removal efficiency for Cd^2+^ in aqueous solution. The adsorption kinetics confirmed to the pseudo-second-order model, while the adsorption isotherm results aligned with the Langmuir model, which collectively suggests that the adsorption process was chemical and monolayer in nature. Comparative XPS and FT-IR analyses of TACA-APTES-SF and TACA-APTES-SF-Cd indicated that adsorption mechanisms involved electrostatic and coordination reactions between hydroxyl-, amino-, and sulfur-containing groups and Cd^2+^ ions. This study therefore proposes a straightforward and cost-effective approach for the high-value utilization of SF.

## 1. Introduction

Cadmium (Cd) primarily originates from industrial activities such as pesticide application, electroplating, tanning, and various chemical manufacturing processes [1,2,3,4], which has led to its persistence as a widespread environmental contaminant. The toxicity of Cd^2+^ is profoundly detrimental, inciting severe health complications including pulmonary edema, renal dysfunction, pneumonia, and oncogenesis even at trace concentrations [4,5,6]. In response to these significant health threats, the World Health Organization (WHO) and United States Environmental Protection Agency (US EPA) have instituted strict guidelines, stipulating that permissible Cd^2+^ concentrations in aqueous environments must be maintained within the narrow range of 0.004 to 0.01 mg·L^−1^. Like other heavy metals, Cd exhibits high environmental persistence, resisting degradation and progressively accumulating within both biotic and abiotic compartments over time [7,8]. Consequently, this non-degradability underscores the critical necessity for efficient removal of Cd^2+^ ions from industrial effluents.

Various methodologies have been applied for the removal of Cd^2+^ ions, including chemical precipitation, ion exchange, membrane filtration, adsorption, and solvent extraction [9]. Among these techniques, adsorption has emerged as one of the most promising for effectively removing Cd^2+^, particularly at low concentrations, owing to its high efficiency, operational simplicity, and versatility. The effectiveness of the adsorption process is highly dependent on the selected adsorbent material. Consequently, a wide range of adsorbents such as magnetite nanoparticles, mesoporous silicon dioxide, carbon nanotubes, and various bio-adsorbents have been developed [2,3,7,10,11,12,13,14]. These materials demonstrate superior properties, including elevated specific surface area and adjustable pore architecture, which enhance their ability to sequester Cd^2+^ ions from aqueous solutions. However, the high cost of raw materials for synthesizing these adsorbents elevates the overall production cost, which limits the large-scale application of adsorption technology. Consequently, the utilization of solid wastes as low-cost raw materials for producing adsorbents has become a major research focus [2,15,16].

SF is a primary solid waste resulting from the manufacturing processes of silicon metal and silicon alloys [17,18]. It is estimated that the production of one ton of silicon metal generates at least 200 kg of SF, leading to an annual output exceeding 500,000 tons in China alone [18]. The extensive generation and subsequent disposal of SF pose significant environmental challenges, contributing to degradation of ecosystems and resulting in the considerable waste of valuable silicon resources. Therefore, there is an urgent need to develop sustainable management strategies for SF to mitigate its adverse environmental impacts. Till now, the recycling of SF has predominantly focused on its incorporation into concrete formulations, where it serves to enhance the mechanical properties and workability of the material due to its significant pozzolanic activity and elevated amorphous silica content [19]. However, recent research has increasingly focused on exploring novel, high-value-added applications for SF. SF typically contains over 85% silicon dioxide, which provides a solid foundation for preparing silicon-based adsorbents [20]. Zhu et al. [21], Chen et al. [22], and their colleagues successfully utilized SF as a silicon source to develop silicon-based adsorbents that exhibited excellent adsorption capacity for lead ions in aqueous solutions. However, the conventional synthesis of silicon-based materials from SF typically involves steps such as impurity removal, purification, dissolution, and reactions under high-temperature and high-pressure conditions. These processes disrupt the inherent microsphere structure of SF, thereby increasing the complexity of the preparation route and the overall production cost.

In contrast, based on the high silicon dioxide content and the inherent microsphere structure of SF, this study proposes a novel strategy to restore surface hydroxyl functional groups through sodium hydroxide activation while preserving the microsphere structure. The surface functionalization is achieved by grafting with silane coupling agents. This approach significantly shortens the preparation process and reduces production costs. This strategy provides a new, low-cost, and high-value pathway for the utilization of SF.

## 2. Results and Discussion

### 2.1. Characterization of the Materials

#### 2.1.1. Scanning Electron Microscopy (SEM) Analysis

**Figure 1:**
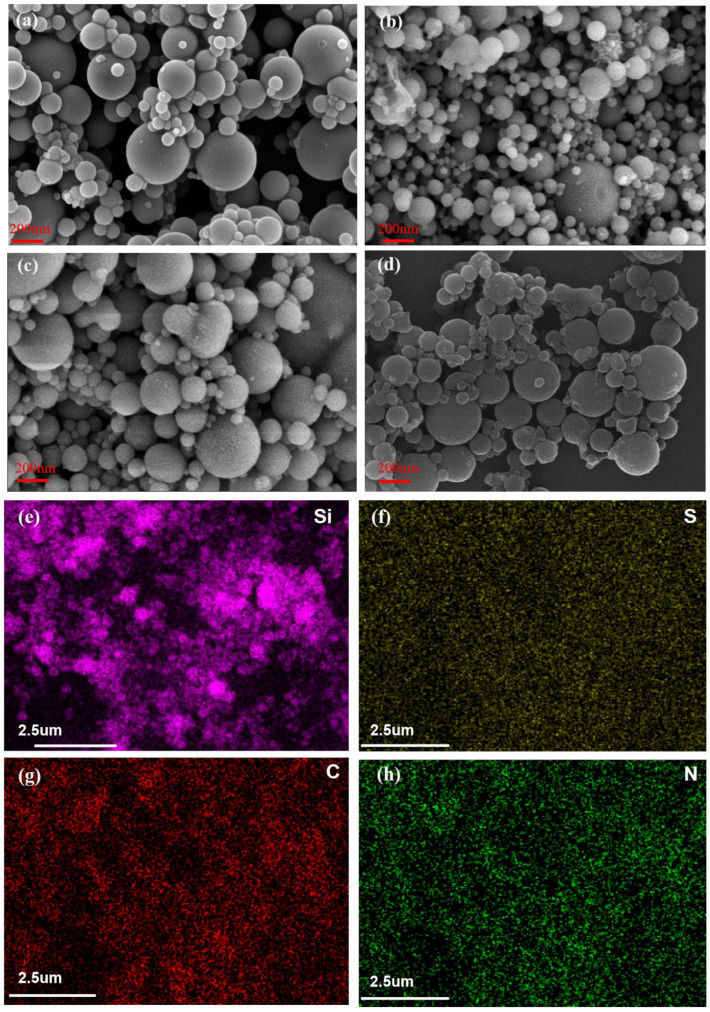
(**a**–**d**) SEM images of SF, A-SF, APTES-SF, and TACA-APTES-SF. (**e**–**h**) SEM mapping of TACA-APTES-SF.

The SEM images of SF, A-SF, APTES, and TACA-APTES-SF are presented in Figure 1a–d, respectively. As shown in Figure 1a, the pristine SF particles exhibited a smooth spherical morphology. In contrast, after calcination and activation, the particle surface became rougher (Figure 1b), indicating that the pretreatment process disrupted the glassy surface layer of the SF. After subsequent modifications (Figure 1c,d), the surface roughness remained pronounced. Notably, no significant change in particle size was observed before and after the functionalization.

Simultaneously, elemental mapping analysis of TACA-APTES-SF was performed. The results (Figure 1e–h) confirmed the homogeneous distribution of nitrogen (N) and sulfur (S) on the material’s surface. Quantitative energy-dispersive X-ray spectroscopy (EDS) analysis revealed surface contents of 3.2 at% for N and 1.8 at% for S, confirming the successful grafting of organic functional groups.

#### 2.1.2. Fourier-Transform Infrared (FT-IR) Spectroscopy

**Figure 2:**
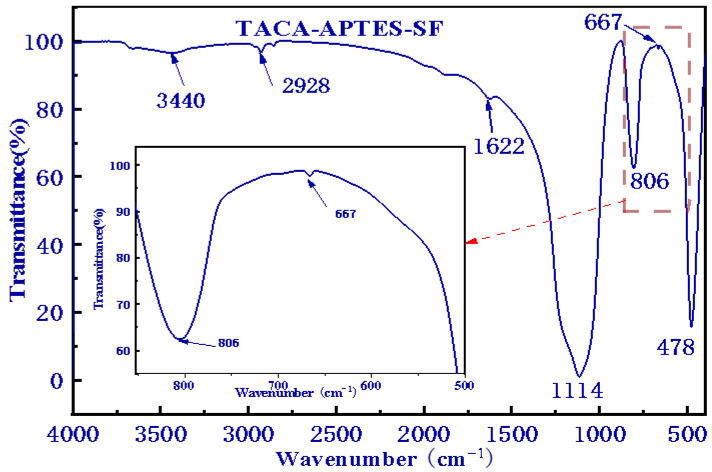
FTIR spectra of TACA-APTES-SF.

**Figure 3:**
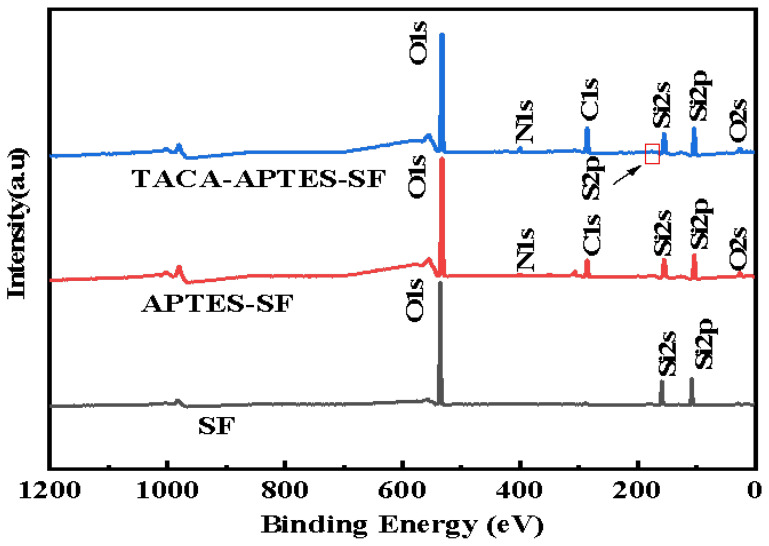
XPS wide-scan spectra of SF, APTES-SF, and TACA-APTES-SF.

The TACA-APTES-SF FT-IR spectra, spanning the range from 400 cm^−1^ to 4000 cm^−1^, are illustrated in Figure 2. The absorption band at 478 cm^−1^ represented bending vibrations of the Si-O-Si bond. The symmetric stretching vibrations of Si-O-Si were identified at 806 cm^−1^ and 1114 cm^−1^ [23]. The peak observed near 2928 cm^−1^ corresponded to C-H stretching vibrations [24,25]. The broad band at 3440 cm^−1^ and the peak at 1622 cm^−1^ were primarily attributed to O-H stretching and H-O-H bending of adsorbed water molecules, respectively [26]. A magnification of the 500–850 cm^−1^ region revealed a low-intensity band at 667 cm^−1^, which may be associated with C-S stretching vibrations [3,27]. It should be noted that definitive confirmation of sulfur incorporation was obtained from XPS analysis (Figure 3). Additionally, due to the spectral overlap between the N-H bending vibration and the band of adsorbed water in FTIR analysis, XPS was employed to provide evidence for the successful grafting of amino groups (Figure 3). Collectively, these FT-IR spectral results demonstrated the successful attachment of target groups to the surface of the SF, thereby confirming the modification process.

#### 2.1.3. X-Ray Photoelectron Spectroscopy (XPS) Analysis

XPS was utilized to characterize the chemical compositions of SF, APTES-SF, and TACA-APTES-SF, and the survey spectra are illustrated in Figure 3. The results indicated that prior to surface modification, the SF exhibited prominent peaks corresponding to O1s, Si2s and Si2p, consistent with its silica-rich composition. Notably, following the surface modification, new peaks attributed to N1s and S2p emerged at approximately 400 eV and 160 eV, respectively, indicating successful grafting of target groups onto SF, consistent with the FT-IR analysis results.

To quantitatively assess the surface composition changes, the elemental atomic percentages (at%) derived from high-resolution XPS analysis are summarized in Table S1. The data clearly indicate a significant increase in nitrogen (N) and sulfur (S) content after functionalization. Specifically, the N content rose from undetectable levels in SF to 3.35 at% in TACA-APTES-SF, while S content reached 0.63 at% in the final material. Concurrently, the relative atomic percentage of silicon (Si) decreased from 29.57 at% in SF to 24.84 at% in TACA-APTES-SF. This decrease can be attributed to the coverage of the silica surface by grafted organic layers, which attenuates the photoelectron signal from underlying Si atoms. The appearance of N and S peaks, coupled with the quantitative data, confirms the successful introduction of functional groups.

#### 2.1.4. Thermogravimetric Analysis (TGA)

**Figure 4:**
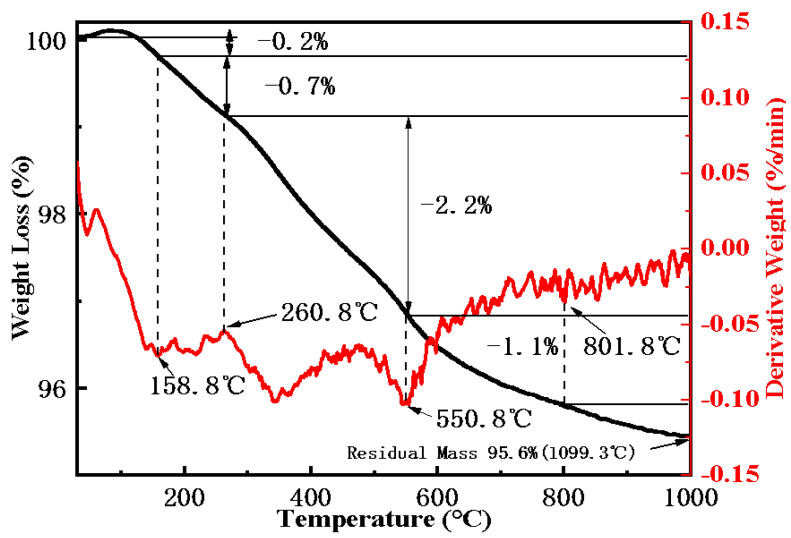
Thermogravimetric analysis of TACA-APTES-SF (30–1100 °C, 10 °C/min, CO_2_ + O_2_).

TGA was conducted to examine the thermal stability of TACA-APTS-SF, revealing the presence of organic molecules. The TGA and DGT curves are presented in Figure 4. The result revealed sequential weight losses of 0.2% (30–158.8 °C), 0.7% (158.8–260.8 °C), 2.2% (260.8–550.8 °C), 1.1% (550.8–801.8 °C), and 0.2% (801.8–1100 °C), giving a total mass loss of 4.4% and a residual mass of 95.6% at 1100 °C. The first two episodes correspond to desorption of residual moisture and scission of surface imine linkages with early oxidation of the 1,3-thiazole ring. The major 2.2% loss between 260.8 and 550.8 °C is attributed to oxidative decomposition of the propyl tether and continued sulfur-oxide evolution. Combustion of residual carbon and desorption of sulfate species account for the 1.1% loss at 550.8–801.8 °C, while the final 0.2% above 800 °C reflects condensation of surface Si–OH, confirming the successful grafting of functional groups onto the surface.

#### 2.1.5. The N_2_ Adsorption–Desorption Isotherms

**Figure 5:**
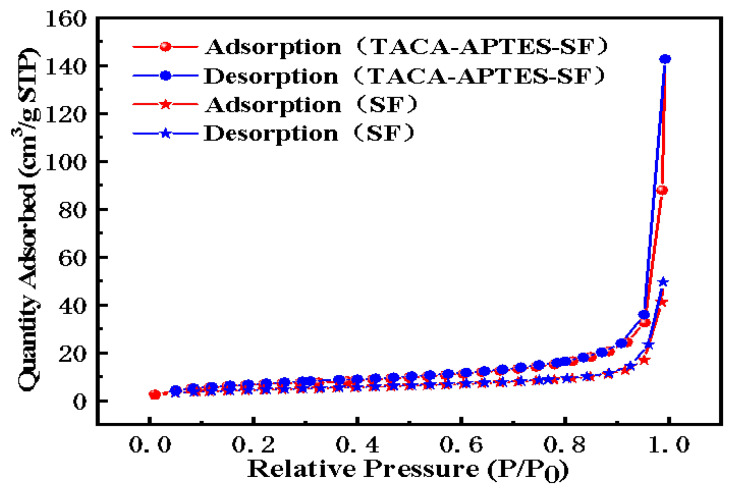
Nitrogen adsorption–desorption isotherms of SF and TACA-APTES-SF.

The N_2_ adsorption–desorption isotherms of SF and TACA-APTES-SF are illustrated in Figure 5. According to the IUPAC classification, both SF and TACA-APTES-SF exhibited type II adsorption isotherms, indicative of non-porous solid characteristics [28,29]. The non-porous nature of SF was primarily attributed to its rapid cooling process during production. In this experiment, functionalization was achieved through surface activation and direct grafting of functional groups without compromising the structural integrity; thus, TACA-APTES-SF retained its non-porous solid characteristics. The specific surface areas were determined to be 16.5 m^2^·g^−1^ for SF and 26.3 m^2^·g^−1^ for TACA-APTES-SF. TACA-APTES-SF possessed superior porosity parameters relative to SF, with pore volume increasing from 0.1 cm^3^·g^−1^ to 0.2 cm^3^·g^−1^ and mean pore diameter expanding from 18.7 nm to 33.6 nm. The increase in specific surface area and pore volume after functionalization is likely due to the alteration of surface roughness and interparticle spacing caused by the grafting of organic molecules, which changes the packing of the primary SF microspheres. This enhanced porosity facilitates greater accessibility to active sites, thereby potentially improving the adsorption capacity for Cd^2+^ ions.

### 2.2. Adsorption Properties

#### 2.2.1. Effect of pH

**Figure 6:**
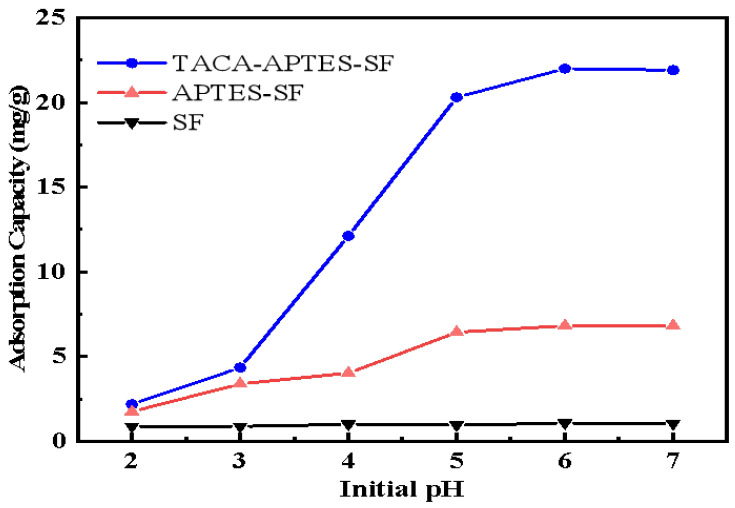
Effect of pH on Cd^2+^ adsorption by TACA-APTES-SF, APTES-SF, and SF (Cd^2+^ concentration was 50 mg·L^−1^, contact time was 120 min, temperature was 25 °C).

In aqueous solutions, pH is a critical factor adjusting the surface charge of material and changing formation of the Cd^2+^ ions [26]. Consequently, the pH value emerges as a significant determinant of adsorption behavior. The results about varying initial pH values influencing adsorption of Cd^2+^ by TACA-APTES-SF, APTES-SF, and SF are presented in Figure 6. Notably, TACA-APTES-SF exhibited a superior adsorption capacity relative to both APTES-SF and SF, suggesting that the newly introduced functional groups effectively enhanced the binding of Cd^2+^ ions.

The adsorption capacity of Cd^2+^ exhibited a marked rise when the pH was elevated from 2 to 7, achieving its maximum at pH 6. This behavior can be explained by the fact that at lower pH levels, the organic functional groups of the surface protonated primarily, which acquired a heightened concentration of hydrogen ions that effectively competed with Cd^2+^ ions for available binding sites [30], thus diminishing the removal capacity.

As the pH increases, the degree of protonation decreases due to the deprotonation of the functional groups. This deprotonation significantly augmented the negative surface charge of the adsorbent, thereby enhancing its capacity for Cd^2+^ adsorption. At pH levels exceeding 7, Cd^2+^ ions primarily existed as Cd(OH)_3_^−^, Cd(OH)^+^, and Cd(OH)_2_ species [12], which could potentially lead to the formation of precipitates. Consequently, to prevent the formation of precipitates that could interfere with the experimental results, all subsequent studies were conducted under a controlled pH of 6.

#### 2.2.2. Adsorption Isotherms

**Figure 7:**
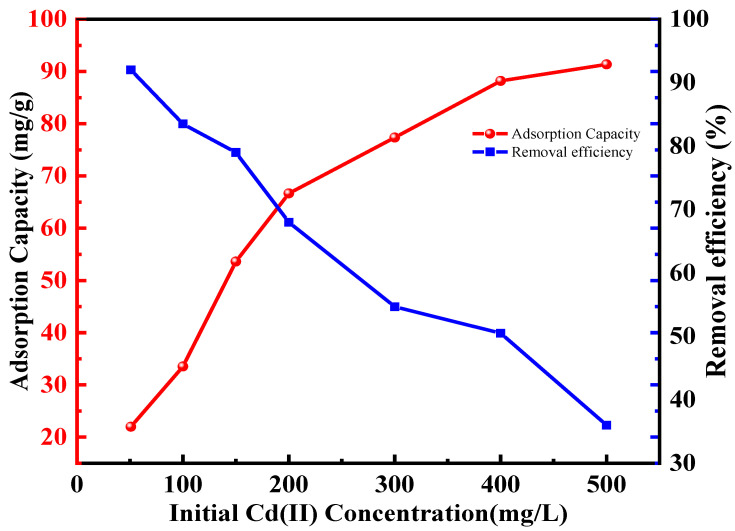
Effect of initial concentration on the Cd^2+^ adsorption capacity (pH value was 6, contact time was 120 min, temperature was 25 °C).

The relationship between the Cd^2+^ initial concentration and the sorption capacity is depicted in Figure 7. It was observed that as Cd^2+^ concentration augmented, the adsorption capacity exhibited a corresponding increase. However, the rate of this enhancement gradually diminished, ultimately achieving a state of equilibrium at 500 mg·L^−1^. The reason for this is that at lower concentrations, the abundant amino and thio groups on the adsorbent facilitated strong interactions, which effectively surpassed the mass transfer resistance between the adsorbent and the aqueous phase. As the active sites were progressively occupied, sorption reached a saturation point, culminating in a maximum of 91.37 mg·g^−1^. This demonstrated that TACA-APTES-SF served as an excellent adsorbent for the adsorption of Cd^2+^ ions, as reflected in Table 1. It was also observed that the removal efficiency decreased with increasing initial Cd^2+^ concentration.

To further elucidate the adsorption mechanism, the experimental data were analyzed using the Langmuir and Freundlich isotherm models. The Langmuir model presupposes that the adsorption process occurs through single-layer coverage on a homogeneous surface, and establishes a dynamic equilibrium between adsorption and desorption processes. Its nonlinear expression is detailed in Equation (1) [36], where Q_f_ representes the equilibrium adsorption capacity, C_f_ denotes the equilibrium concentration of Cd^2+^, and K_L_ is the Langmuir adsorption constant. The parameter Qm (mg·g^−1^) signifies the maximum capacity of adsorption for Cd^2+^ as predicted by the Langmuir model.

(1) $$Q_{f}=\frac{Q_{m}\cdot K_{L}\cdot C_{f}}{1+K_{L}\cdot C_{f}}$$

The Freundlich isotherm model, recognized as an empirical equation, can be articulated in its nonlinear form as delineated in Equation (2) [37]. This model posits that the adsorption mechanism is characterized by multi-layer adsorption onto irregular surfaces, thereby indicating that the sorption process is reversible and not ideal, with significant interactions among the sorbed molecules. Within this framework, the parameters K_F_ and n serve as constants that encapsulate the distinctive adsorption characteristics of the system.

(2) $$Q_{f}=K_{F}\cdot {C_{f}}^{\frac{1}{n}}$$

**Figure 8:**
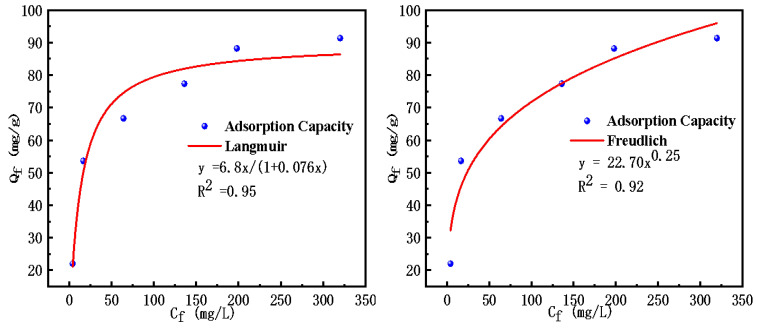
Adsorption isotherm curves of Cd^2+^ on TACA-APTES-SF.

The results of the fitting analysis concerning the Langmuir and Freundlich adsorption isotherm models are illustrated in Figure 8. Additionally, the associated fitting parameters, along with their respective correlation coefficients, are summarized in Table 2. The analysis revealed that the Langmuir model displayed a markedly superior correlation coefficient (R^2^ = 0.95) in comparison to the Freundlich isotherm model (R^2^ = 0.92), thereby indicating that the Langmuir model offered a more precise characterization of the sorption process. Furthermore, the computed maximum adsorption capacity of 89.94 mg·g^−1^ exhibited a close correspondence with the empirical result of 91.37 mg·g^−1^. These observations implied that the surface characteristics of TACA-APTES-SF were homogeneous and the adsorption mechanism predominantly adhered to a monolayer adsorption framework.

The slight deviation (1.6%) between the Langmuir-predicted Qm and the experimental value may be attributed to minor multilayer adsorption at high concentrations, which is consistent with the Freundlich exponent n > 1.

In this study, the favorability of the adsorption process was assessed utilizing the dimensionless separation factor (K_1_), which is derived from the Langmuir model. The value of K_1_ holds significant implications for the characterization of the adsorption process: a K_1_ value of 0 indicates that the adsorption procedure is deemed irreversible, while a K_1_ value within the range of 0 to 1 suggests that the process is favorable. Conversely, a K_1_ value of 1 classifies the adsorption behavior as linear, and a K_1_ value greater than 1 implies that the process is unfavorable. The mathematical expression for K_1_ can be found in Equation (3) [38], providing a quantitative framework for evaluating the efficiency and reversibility of the adsorption phenomena under consideration.

(3) $$K_{1}=\frac{1}{1+K_{L}\cdot C_{i}}$$

where K_L_ represents the Langmuir constant, while C_i_ denotes the initial concentration of Cd^2+^.

The calculated K_1_ values for different initial Cd^2+^ concentrations are detailed in Table 3. It was evident that all calculated values of K_1_ fell within the range of 0 to 1, and the K_1_ value decreased with increasing initial Cd^2+^ concentration. These results confirm that the adsorption process is favorable under the studied conditions.

#### 2.2.3. Adsorption Kinetics

In general, as the adsorption process advances, there is a progressive occupation of active sites on the adsorbent’s surface, which ultimately leads to a decline in adsorption efficiency. Consequently, the duration of the adsorption reaction emerges as a critical variable influencing the overall adsorption dynamics.

**Figure 9:**
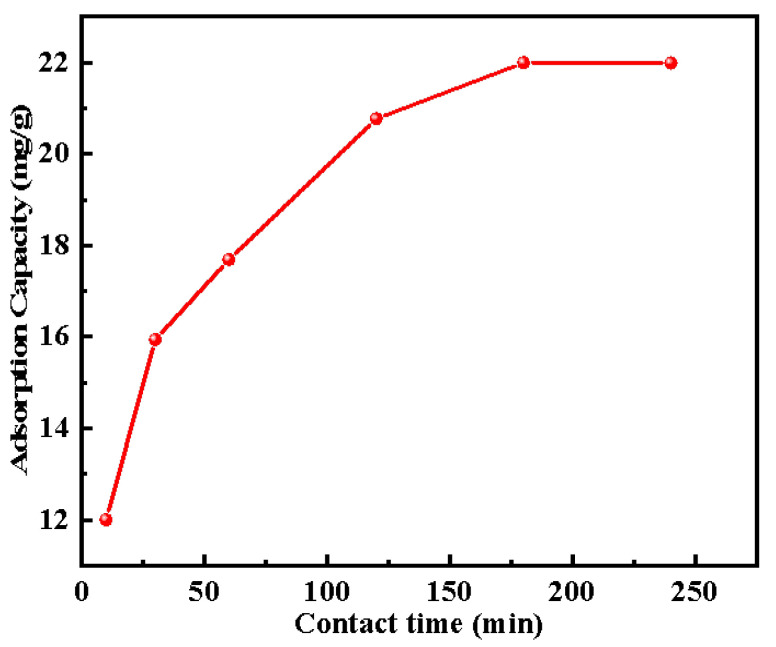
Effect of the contact time on the adsorption capacity of Cd^2+^ by TACA-APTES-SF (pH value was 6, Cd^2+^ concentration was 50 mg·L^−1^, temperature was 25 °C).

The effects of contact time on the adsorption capacity of Cd^2+^ using TACA-APTES-SF are illustrated in Figure 9. Initially, the capacity of adsorption exhibited a pronounced increase; however, after 60 min, the rate of change diminished, ultimately reaching equilibrium at 180 min. The initial significant rise was ascribed to the abundance of surface active sites. The subsequent slowdown was due to the saturation of these sites, which limits further Cd^2+^ uptake.

To elucidate the mechanisms occurring at different adsorption times, the kinetic data were fitted with the pseudo-first-order model (Equation (4)) and the pseudo-second-order model (Equation (5)). The pseudo-first-order model posits that the occupation of adsorption sites occurs in direct proportion to both the driving force and the quantity of unoccupied sites, thereby primarily reflecting physical adsorption phenomena [39]. In contrast, the pseudo-second-order model is based on the quantity of adsorbate present on the solid phase and suggests that chemical adsorption constitutes the dominant mechanism, with the rate of site occupation being proportional to the square of the number of unoccupied sites [40].

(4) $$Ln(Q_{f}-Q_{t})=LnQ_{f}-k_{1}t$$

(5) $$Q_{t}=\frac{{Q_{f}}^{2}k_{2}t}{1+Q_{f}k_{2}t}$$

where Q_f_ (mg·g^−1^) primarily describes the adsorption capacity at the equilibrium time, Q_t_ (mg·g^−1^) depicts the adsorption capacity at any given time, and the rate constants of pseudo-first-order and pseudo-second-order models are k_1_ and k_2,_ respectively.

**Figure 10:**
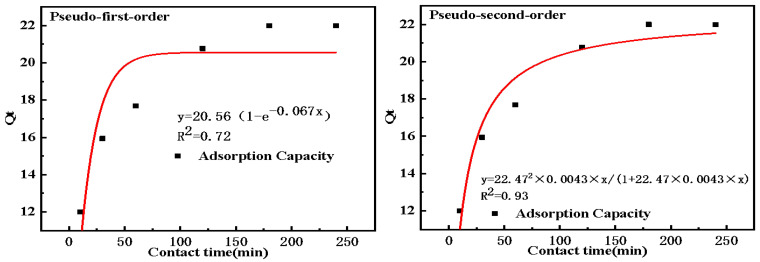
Fitting curve of adsorption kinetic models of Cd^2+^ on TACA-APTES-SF.

The kinetic data fitted with both kinetic models are presented in Figure 10, and the corresponding parameters are summarized in Table 4. A comparison of two kinetic results showed that the correlation coefficient of the pseudo-second-order kinetic model (R^2^ = 0.93) was substantially higher than the pseudo-first-order kinetic model (R^2^ = 0.72). Moreover, the Q_f_ (22.47 mg·g^−1^) obtained under the pseudo-second-order kinetic model closely matched the experimental value (22.00 mg·g^−1^). Therefore, it can be inferred that the adsorption behavior was effectively characterized by the pseudo-second-order kinetic model, thereby exhibiting that the chemical adsorption was the primary mechanism governing the interaction between active sites and Cd^2+^ ions.

#### 2.2.4. Reusability

**Figure 11:**
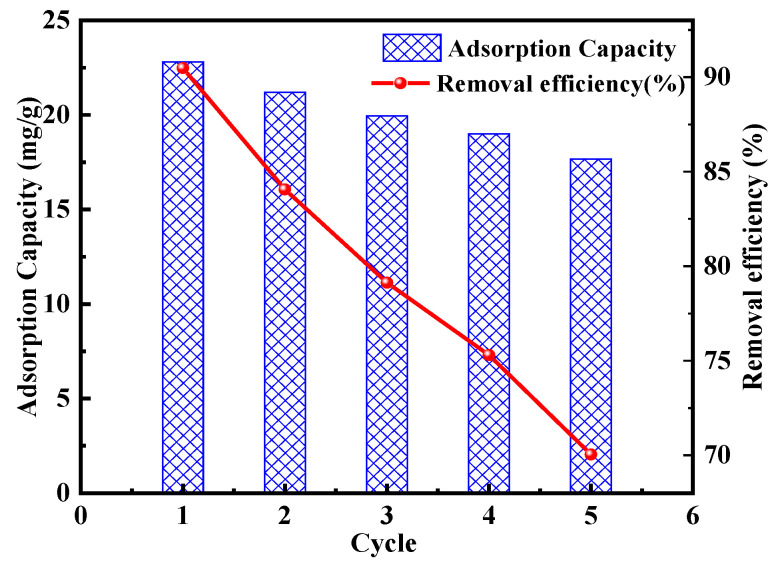
Effect of TACA-APTES-SF regeneration on Cd^2+^ adsorption capacity.

In pursuit of resource conservation and environmental sustainability, the adsorption and desorption processes were conducted over five cycles. As illustrated in Figure 11, the adsorption performance for Cd^2+^ remained substantial, with a reduction in removal efficiency of only 22.6% after five cycles. The removal efficiency declined was due to the mass loss of TACA-APTES-SF during the adsorption–desorption cycles. Overall, TACA-APTES-SF exhibits good stability and reusability for Cd^2+^ removal.

### 2.3. Adsorption Mechanism

**Figure 12:**
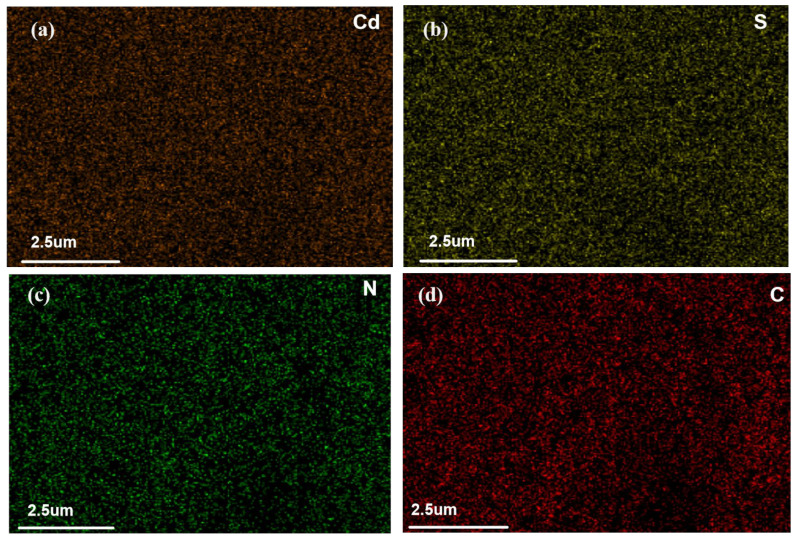
SEM mapping after adsorption of Cd^2+^.

SEM-EDS mapping was used to analyze the distribution of elements on TACA-APTES-SF after Cd^2+^ adsorption. The corresponding elemental maps are shown in Figure 12: (a) confirms the presence of cadmium distributed on the adsorbent surface after adsorption; (b) and (c) indicate a decrease in the intensity of the S and N signals compared to the pre-adsorption state, suggesting that the corresponding functional groups are involved in binding Cd^2+^ ions; (d) clearly shows the presence and uniform distribution of carbon throughout the material. This indicates that N- and S-containing functional groups play a significant role in the adsorption process.

**Figure 13:**
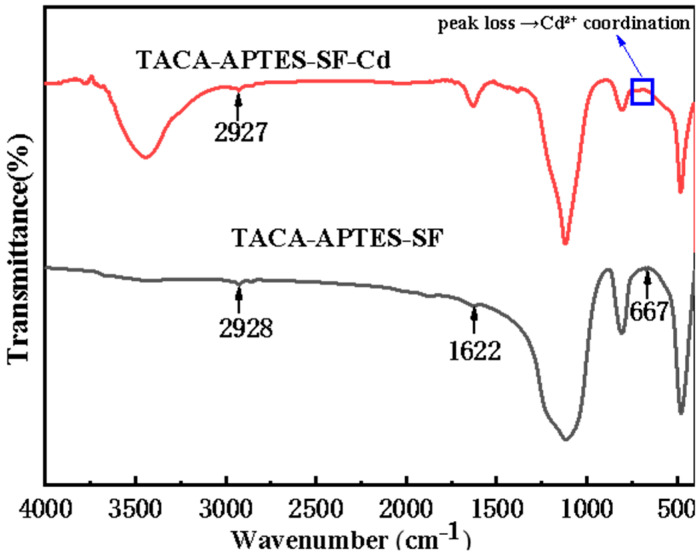
FT-IR spectra of TACA-APTES-SF and TACA-APTES-SF-Cd.

**Figure 14:**
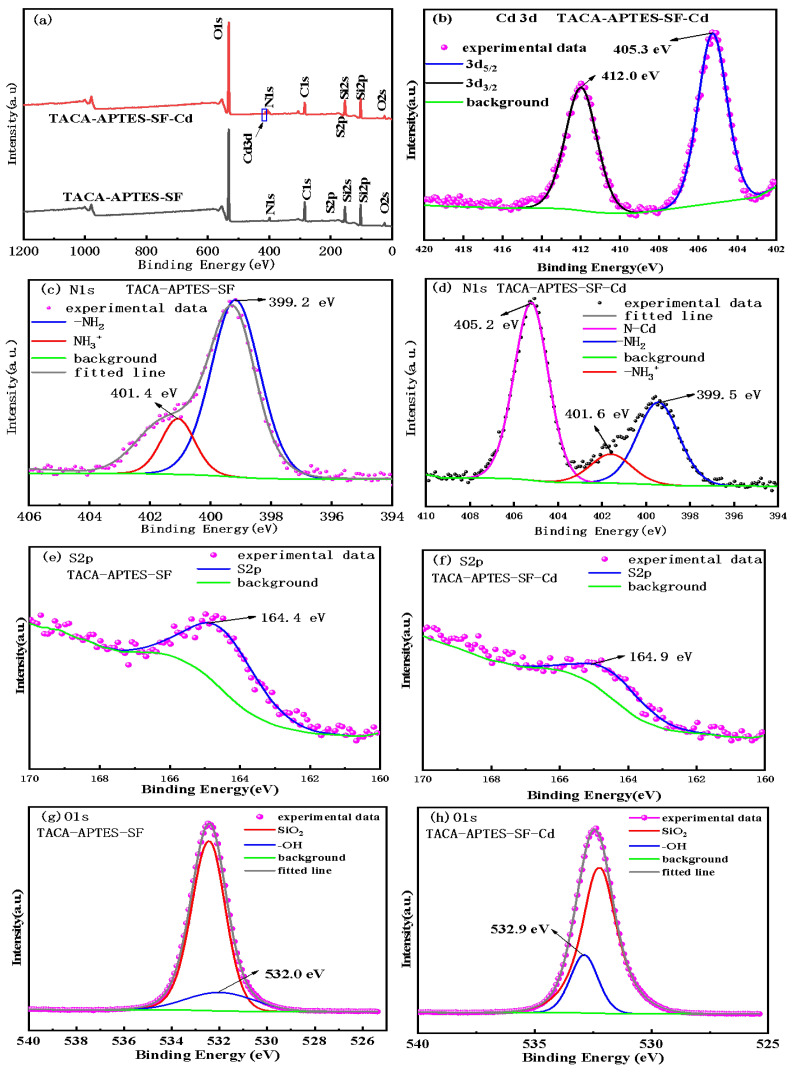
XPS wide-scan spectrum of TACA-APTES-SF and TACA-APTES-SF-Cd (**a**); high-resolution spectra of Cd3d of TACA-APTES-SF-Cd (**b**); high-resolution spectra of N1s of TACA-APTES-SF (**c**); high-resolution spectra of N1s of TACA-APTES-SF-Cd (**d**); high-resolution spectra of S2p of TACA-APTES-SF (**e**); high-resolution spectra of S2p of TACA-APTES-SF-Cd (**f**); high-resolution spectra of O1s of TACA-APTES-SF (**g**); high-resolution spectra of O1s of TACA-APTES-SF-Cd (**h**).

The FT-IR spectra of TACA-APTES-SF, and TACA-APTES-SF-Cd are presented in Figure 13. No significant shift in C-H vibration (2928→2927 cm^−1^) was observed, while the disappearance of the peak at ~667 cm^−1^ (assigned to C-S) in conjunction with the positive shift in the S2p binding energy observed in XPS (from 164.4 eV to 164.9 eV, Figure 14f) collectively suggests that the sulfur atom in the thiazole ring participates in coordination with Cd^2+^ ions [1,40]. The change in electron density around the S atom upon coordination alters its vibrational signature and increases its core-level binding energy.

The chemical states of the elements in TACA-APTES-SF, and TACA-APTES-SF-Cd were further analyzed by high-resolution XPS, as shown in Figure 14. The survey spectrum of TACA-APTES-SF-Cd (Figure 14a) clearly shows the presence of Cd3d peaks, confirming the adsorption of Cd^2+^. The high-resolution Cd 3d spectrum (Figure 14b) shows doublet peaks at binding energies of 405.3 eV (Cd 3d_5_/_2_) and 412.0 eV (Cd 3d_3_/_2_), confirming the presence of Cd^2+^.

In the N1s high-resolution spectra (Figure 14c), TACA-APTES-SF exhibited peaks of 399.2 eV and 401.4 eV, corresponding to pyridinic (N-6) and -NH_2_, respectively [41]. After Cd^2+^ adsorption (TACA-APTES-SF-Cd), the -NH_2_ peak shifted to 401.6 eV, the pyridinic (N-6) peak shifted to 399.5 eV, and a new peak attributed to N-Cd coordination appeared at 405.2 eV. The S2p spectrum of TACA-APTES-SF showed a peak at 164.4 eV, which shifted to 164.9 eV after Cd^2+^ adsorption (TACA-APTES-SF-Cd). The positive shifts in the N1s and S2p binding energies after adsorption are primarily due to coordination between the N/S atoms and Cd^2+^, which reduces the electron density on the N and S atoms [42,43,44].

Furthermore, the binding energies of O1s peaks before and after adsorption (Figure 14g,h) revealed a noticeable increase in the binding energy of the hydroxyl group (−OH), shifting from 532.0 eV to 532.9 eV. This finding indicated the participation of −OH in the adsorption process of Cd^2+^. However, a critical observation from the adsorption isotherm data indicates that the maximum adsorption capacity for Cd^2+^ reached 91.37 mg·g^−1^ (approximately 814 μmol·g^−1^), which is substantially higher than the estimated grafted functional group density (~287 μmol·g^−1^ from TGA and XPS analysis). This discrepancy suggests that the native silanol groups (-Si-OH) on the silica fume surface play a predominant role in the adsorption process, likely through ion exchange or electrostatic interactions, in addition to the coordination effects of the grafted -NH_2_ and -S groups. The measurable shift in the O1s binding energy (from 532.0 eV to 532.9 eV) further supports the involvement of silanol groups in Cd^2+^ coordination, as the electron density around oxygen atoms decreases upon metal ion binding.

Collectively, these results suggested that nitrogen, oxygen, and sulfur atoms act as electron donors in the adsorption of Cd^2+^, facilitating the formation of coordination complexes. Thus, it can be concluded that the adsorption mechanism involves a synergistic effect where both grafted functional groups (-NH_2_, -S) and native silanol groups (-Si-OH) contribute to Cd^2+^ uptake, with the latter providing the primary adsorption capacity due to their abundance on the silica surface.

**Figure 15:**
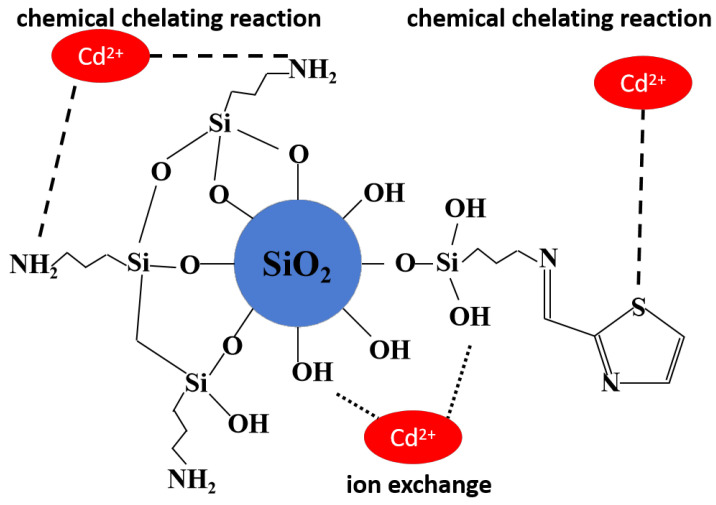
The adsorption mechanism of Cd^2+^ on TACA-APTES-SF.

Consequently, the 0.5 eV shift in S2p, the appearance of the N-Cd peak, and the O1s shift collectively provide evidence for coordination bonding between Cd^2+^ and the N, S, and O atoms [42]. The synergy of -NH_2_, thiazole-S, and -OH enabled multi-dentate chelation (Figure 15).

## 3. Materials and Methods

### 3.1. Materials and Reagents

The SF utilized was sourced from a local metallurgy-grade silicon manufacturing facility. 3-aminopropyltriethoxysilane (APTES, purity > 98.5%) was acquired from West Asia Chemical Industry Co., Ltd. (Linyi, China), while 1,3-Thiazole-2-carbaldehyde (TACA, purity > 98%) was obtained from Bide Pharmatech Co., Ltd. (Shanghai, China). Additional reagents, including NaOH (99%), ethanol (99.8%), HNO_3_ (67%), and acetic acid (99.8%), were provided by Nanjing Chemical Reagent Co., Ltd. (Nanjing, China). Cadmium nitrate (Cd(NO_3_)_2_) was sourced from the National Nonferrous Metals Research Institute (Beijing, China). pH adjustments were accomplished employing HNO_3_ and NaOH solutions (0.1 M).

### 3.2. Pretreatment of SF

**Figure 16:**
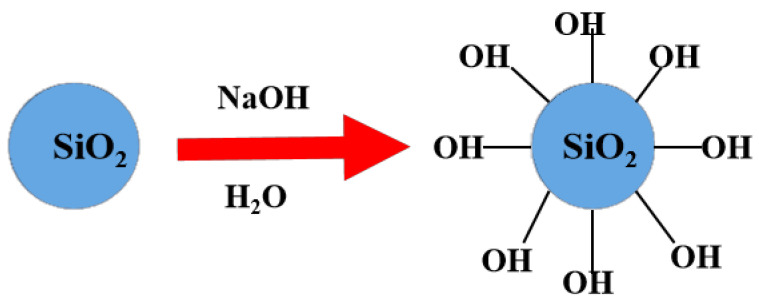
Activation of the SF.

As-produced SF typically possesses a limited number of surface hydroxyl groups (-OH) due to the high-temperature rapid cooling process during its formation [45]. Furthermore, about 1–5% carbon was contained in the SF, decreasing the silica content. Therefore, before the synthesis began, SF was manufactured by calcination and activation (Figure 16).

A muffle furnace was utilized to calcine the SF at 550 °C for a duration of 6 h, after which the material was allowed to cool to ambient temperature. Subsequently, a total of 4 g of the calcined SF was suspended in 250 mL of a 0.1 M aqueous sodium hydroxide solution [46]. The activation conditions (0.1 M NaOH, 65 °C, 24 h) were carefully optimized to achieve sufficient surface hydroxylation while preventing significant dissolution of silica that could lead to gelation or degradation of the particulate structure. The resulting mixture was stirred in a water bath at a controlled temperature of 65 °C for 24 h, employing a magnetic stirrer. The preservation of the spherical morphology throughout this activation step was confirmed by SEM analysis (Figure 1b), indicating that the treatment effectively generated surface silanol groups without compromising the microsphere integrity.

Following this treatment, the mixture was neutralized with acetic acid before subjecting it to centrifugation. The precipitate obtained was then dried at 85 °C for an additional 24 h. The resulting SF will henceforth be designated as A-SF.

### 3.3. Synthesis of TACA-APTES-SF

**Figure 17:**
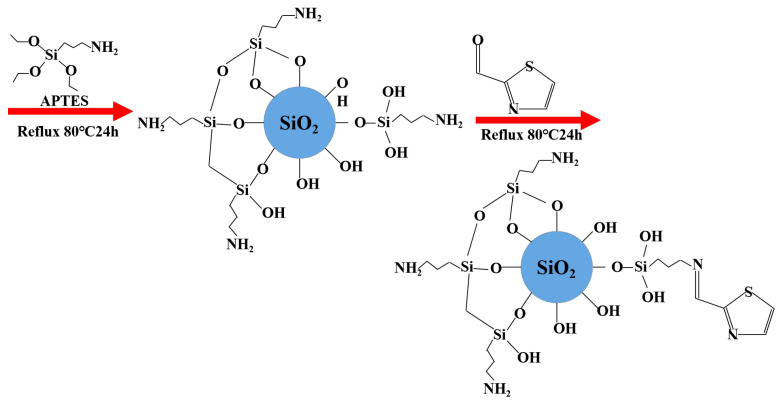
Synthesis route of the TACA-APTES-SF.

The synthesis pathway for TACA-APTES-SF is depicted in Figure 17. Initially, 5 g A-SF was suspended in 100 mL ethanol. Subsequently, 10 mL of APTES was added dropwise to this dispersion, refluxed under a nitrogen protective atmosphere at 80 °C for 24 h. The amount of APTES used was based on the theoretical maximum grafting density of APTES on silica (approximately 2–3 μmol·m^−2^), considering the specific surface area of A-SF (16.5 m^2^·g^−1^). This calculation estimated an amino group loading of approximately 33–50 μmol·g^−1^ for the functionalized material. Following this treatment, the resulting precipitate was recovered and repeatedly washed with ethanol and deionized water in alternating steps to remove any physisorbed reactants, followed by drying under vacuum at 80 °C. The obtained product was designated as APTES-SF.

Subsequently, 4 g APTES-SF was introduced into 100 mL ethanol, and then 10 mL TACA and 1 mL acetic acid were added. The quantity of TACA was determined based on a 1.2:1 molar ratio relative to the estimated amino group loading (33–50 μmol·g^−1^), ensuring a sufficient excess to drive the Schiff base reaction to completion. This reaction mixture was refluxed for an additional 24 h at 80 °C under a nitrogen atmosphere. Upon completion of the reaction, the product was isolated, washed multiple times with ethanol and deionized water to remove any physisorbed reactants, and subjected to vacuum drying at a temperature of 80 °C for 12 h. The final product was referred to as TACA-APTES-SF.

### 3.4. Characterization

The materials’ morphology and elemental compositions were assessed utilizing SEM with a ZEISS Sigma 300 instrument (Jena, Germany). The presence of functional groups within the samples was evaluated through FT-IR using a Thermo Fisher Nicolet iN10 spectrometer (Waltham, MA, USA). The elemental composition of the samples was determined employing XPS with the Thermo Scientific K-Alpha system. Furthermore, an inductively coupled plasma optical emission spectrometer (ICP-OES, PerkinElmer 8300, Waltham, MA, USA) was employed to quantify the concentration of metal ions. The samples underwent thermogravimetric analysis (TGA) by being heated from room temperature to 1100 °C at a rate of 10 °C·min^−1^ under an argon atmosphere (Netzsch TG 209 F1, Selb, Germany). The specific surface area was assessed using the Brunauer–Emmett–Teller (BET) method, utilizing a Micromeritics ASAP 2460 instrument (Norcross, GA, USA) at a temperature of 77 K.

### 3.5. Adsorption Experiments

Batch adsorption experiments were conducted to investigate the effects of initial pH (2–7), contact time (10–240 min), and initial Cd^2+^ concentration on the adsorption performance of TACA-APTES-SF. In a typical experiment, 20 mg of TACA-APTES-SF was added to a 15 mL centrifuge tube containing 10 mL of a Cd^2+^ solution at a known concentration. The mixture was agitated on a shaker for a predetermined time. The sample was centrifuged at 7000 rpm for 15 min, and the supernatant was analyzed.

The pH of the solutions was adjusted to the desired value (2–7) using 0.1 M HNO_3_ and NaOH, and measured with an OHAUS STARTER 3C pH meter (Parsippany, NJ, USA). The adsorption kinetics were studied at pH 6.0, an initial Cd^2+^ concentration of 50 mg·L^−1^, and 25 °C, with sampling at different time intervals. The adsorption isotherms were determined at pH 6.0 and 25 °C, with initial Cd^2+^ concentrations ranging from 50 to 500 mg/L.

In order to evaluate the adsorbent’s reusability, 50 mg of TACA-APTES-SF was immersed in 10 mL of a Cd^2+^ solution at a concentration of 50 mg·L^−1^ and subjected to agitation for a duration of 24 h. After adsorption, the adsorbent was separated by centrifugation. The Cd^2++^-loaded adsorbent was then desorbed by shaking with 10 mL of 1 M HCl for 24 h. After desorption, the adsorbent was rinsed with deionized water until the washings reached neutral pH. This adsorption–desorption cycle was repeated five times.

During the adsorption process, adsorption capacity (Q, mg·g^−1^) and removal efficiency (R,%) are critical indicators that reflect the performance of the adsorbent material. The respective mathematical formulations for both parameters are provided in Equations (6) and (7), respectively.

(6) $$Q=(C_{i}-C_{f})\times \frac{V}{m}$$

(7) $$R=\frac{C_{i}-C_{f}}{C_{i}}\times 100\%$$

where C_f_ (mg·L^−1^) represents the final concentration of Cd^2+^ ions, and C_i_ (mg·L^−1^) denotes the initial concentration of Cd^2+^ ions. Additionally, V (mL) refers to the Cd^2+^ ion solution volume, while m (mg) indicates the TACA-APTES-SF’s mass.

## 4. Conclusions

In this study, a novel adsorbent (TACA-APTES-SF) was successfully synthesized by a straightforward functionalization of SF. The pretreatment involving calcination and activation effectively generated surface silanol groups without destroying the microsphere structure. Most of the adsorption of Cd^2+^ occurred within the initial 60 min, achieving a maximum adsorption capacity of 91.37 mg·g^−1^ for TACA-APTES-SF at 298 K and a pH value of 6. The adsorption process was found to conform closely to Langmuir isotherm and the pseudo-second-order kinetic model, indicating that the underlying mechanism was predominantly characterized by monolayer chemical adsorption. Additionally, the results from five regeneration cycles confirmed the adsorbent’s effective regenerability. This approach not only addresses the environmental risks of SF disposal but also provides a cost-effective solution for heavy metal remediation in industrial wastewater. Future studies should explore the scalability of this method and its adaptability to other heavy metal pollutants.

## Figures and Tables

**Table 1:** Comparative analysis of Cd^2+^ adsorption capacities of various adsorbents reported in the literature.

Adsorbent	Test Parameters		Adsorption Capacity (mg·g^−1^)	References
	Value of pH	Adsorption Equilibrium Duration (min)		
MCM-41	7	180	8.56	[31]
amino-functionalized silica	6	20	59.9	[32]
Bioinspired Mesoporous Silica	6.6	120	116.896	[33]
magnetic nanosilica	6	120	4.11	[34]
silica	5	120	4.8332	[35]
Functional SF	6	120	91.37	This study

**Table 2:** Adsorption isotherm fitting parameters of adsorption of Cd^2+^ by TACA-APTES-SF.

Langmuir isotherm model	R^2^	K_L_	Qm (mg·g^−1^)
	0.95	0.076	89.94
Freundlich isotherm model	R^2^	K_F_	*n*
	0.92	22.70	4.00

**Table 3:** The K_1_ values derived from the Langmuir model.

C_0_ (mg·L^−1^)	50	100	150	200	300	400	500
*K*_1_	0.9515	0.9091	0.8696	0.8333	0.7692	0.7143	0.6667

**Table 4:** Kinetic fitting parameters of adsorption of Cd^2+^ by TACA-APTES-SF.

Pseudo-first-order model	R^2^	k_1_ (1·min^−1^)	Q_f_ (mg·g^−1^)
	0.72	0.067	20.56
Pseudo-second-order model	R^2^	k_2_ (g·mg^−1^·min^−1^)	Q_f_ (mg·g^−1^)
	0.93	0.0043	22.47

## Data Availability

Data are contained within the article.

## Supplementary Materials

The following supporting information can be downloaded at: https://www.mdpi.com/article/10.3390/molecules30204141/s1, Table S1: The elemental atomic percentages (at%); Table S2: Comparative analysis of Cd^2+^ adsorption capacities of various adsorbents reported in the literature; Table S3: Adsorption isotherms fitting parameters of adsorption of Cd^2+^ by TACA-APTES-SF; Table S4: The K1 values derived from the Langmuir model; Table S5: Kinetic fitting parameters of adsorption of Cd^2+^ by TACA-APTES-SF.
