# Supplementary material for: Functionalized Silica Fume for Efficient Cd2+ Removal from Aqueous Solutions
Source: Molecules. 2025 Oct 21;30(20):4141. doi: 10.3390/molecules30204141 (PMC12566601; doi:10.3390/molecules30204141)
Supplement: Supplementary file 1 [file molecules-30-04141-s001.zip › molecules-3898911-supplementary.pdf]

# Functionalized Silica Fume for Efficient $Cd^{2+}$ Removal from Aqueous Solutions

**Jianeng Zhu [1,2] and Kuixian Wei [1,*]**

[1] Faculty of Metallurgical and Energy Engineering, Kunming University of Science and Technology, Kunming 650093, China; zhujn2019@126.com
[2] Kunming General Survey of Natural Resources Center, Kunming 650111, China
* Correspondence: kxwei2008@hotmail.com

## 1. The elemental atomic percentages (at%) of the adsorbent by XPS

**Table S1.** The elemental atomic percentages (at%).

| Sample | Atomic percentages (at%) | | | | |
|---|---|---|---|---|---|
| | Si 2p | O 1s | C 1s | N 1s | S 2p |
| SF | 29.57 | 61.97 | 8.46 | | |
| APTES-AF | 25.17 | 50.70 | 21.16 | 2.97 | |
| TACA-APTES-AF | 24.84 | 44.49 | 26.69 | 3.35 | 0.63 |

## 2. The effect of $Cd^{2+}$concentration and adsorption isotherms

**Table S2.** Comparative analysis of $Cd^{2+}$ adsorption capacities of various adsorbents reported in the literature.

| Adsorbent | Test parameters | | Adsorption capacity (mg/g) | References |
|---|---|---|---|---|
| | value of pH | Adsorption equilibrium duration (min) | | |
| MCM-41 | 7 | 180 | 8.56 | [31] |
| amino-functionalized silica | 6 | 20 | 59.9 | [32] |
| Bioinspired Mesoporous Silica | 6.6 | 120 | 116.896 | [33] |
| magnetic nanosilica | 6 | 120 | 4.11 | [34] |
| silica | 5 | 120 | 4.8332 | [35] |
| Functional SF | 6 | 120 | 91.37 | This study |

**Table S3.** Adsorption isotherms fitting parameters of adsorption of $Cd^{2+}$ by TACA-APTES-SF.

| | | | |
|---|---|---|---|
| Langmuir isotherm model equation | $R^2$ | $K_L$ | Qm（$mg\cdot g^{-1}$） |
| | 0.95 | 0.076 | 89.94 |
| Freundlich isotherm model equation | $R^2$ | $K_F$ | n |
| | 0.92 | 22.70 | 4 |

In the models, $Q_f$, $C_f$ and $K_L$ were the equilibrium adsorption capacity, equilibrium concentration of $Cd^{2+}$ and constant of Langmuir, respectively. Qm (mg/g) was the Langmuir

maximum adsorption capacity of $Cd^{2+}$.$K_F$ and n were the constants.

**Table S4.** The K1 values derived from the Langmuir model.

| $C_0$(mg/L) | 50 | 100 | 150 | 200 | 300 | 400 | 500 |
|---|---|---|---|---|---|---|---|
| $K_R$ | 0.9515 | 0.9091 | 0.8696 | 0.8333 | 0.7692 | 0.7143 | 0.6667 |

The dimensionless separation constant ($K_R$) was applied in this study to predict whether the adsorption was favorable process or unfavorable process. Here, if the value of $K_R$ equaled 0, the adsorption was irreversible case; if the $K_R$ fell within the range of 0 to 1, the adsorption was favorable; if $K_R$ equaled 1, the adsorption was linear case; and if the value of $K_R$ was > 1, the adsorption process was unfavorable.

## 3. The effect of time on adsorption capacity

**Table S5.** Kinetic fitting parameters of adsorption of $Cd^{2+}$ by TACA-APTES-SF.

| | | | |
|---|---|---|---|
| Pseudo-first-order model Equation | $R^2$ | $k_1$(1·min$^{-1}$) | $Q_f$ (mg·g$^{-1}$) |
| | 0. 72 | 0. 067 | 20. 56 |
| Pseudo-second-order model Equation | $R^2$ | $k_2$(g·mg$^{-1}$·min$^{-1}$) | $Q_f$ (mg·g$^{-1}$) |
| | 0. 93 | 0. 0043 | 22.47 |

In the models, Qf (mg/g) and Qt (mg/g) were the adsorption capacity at the equilibrium time and any time, respectively. k1 and k2 were the rate constant of Pseudo-first/second-order , respectively.
